# Geosmin Attracts *Aedes aegypti* Mosquitoes to Oviposition Sites

**DOI:** 10.1016/j.cub.2019.11.002

**Published:** 2019-12-12

**Authors:** Nadia Melo, Gabriella H. Wolff, Andre Luis Costa-da-Silva, Robert Arribas, Merybeth Fernandez Triana, Muriel Gugger, Jeffrey A. Riffell, Matthew DeGennaro, Marcus C. Stensmyr

**Affiliations:** 1 Department of Biology, Lund University, 22362 Lund, Sweden; 2 Department of Biology, University of Washington, Seattle, WA 98195-1800, USA; 3 Department of Biological Sciences & Biomolecular Sciences Institute, Florida International University, Miami, FL 33199, USA; 4 Instituto de Quimica e Biotecnologia, Universidade Federal de Alagoas, Maceio 5702-970, Brazil; 5 Collection of Cyanobacteria, Institut Pasteur, 75015 Paris, France; 6 Lead Contact

## Abstract

Melo et al. show that geosmin mediates egg laying in the yellow fever mosquito *Aedes aegypti*, which associates geosmin with microbes present in the larval aquatic habitat. The authors further show that geosmin can be used as bait in oviposition traps and that geosmin can be substituted by beetroot peel for mosquito trapping in developing countries.

## Graphical Abstract

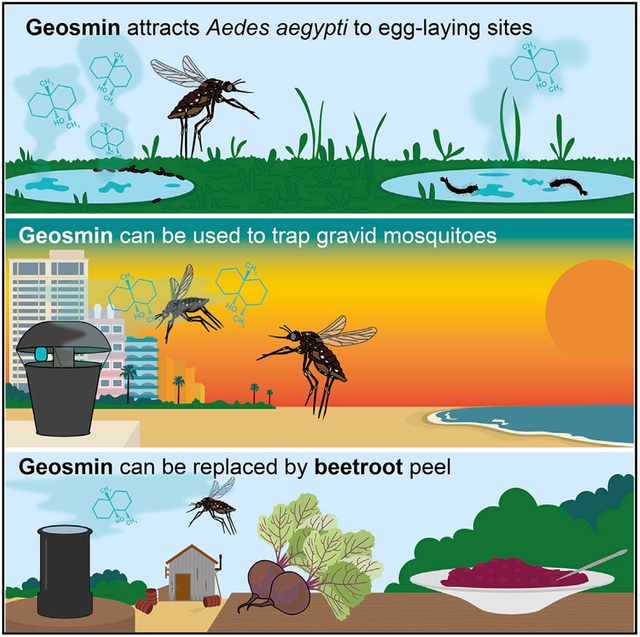

## SUMMARY

Geosmin is one of the most recognizable and common microbial smells on the planet. Some insects, like mosquitoes, require microbial-rich environments for their progeny, whereas for other insects such microbes may prove dangerous. In the vinegar fly *Drosophila melanogaster*, geosmin is decoded in a remarkably precise fashion and induces aversion, presumably signaling the presence of harmful microbes [1]. We have here investigated the effect of geosmin on the behavior of the yellow fever mosquito *Aedes aegypti*. In contrast to flies, geosmin is not aversive but mediates egg-laying site selection. Female mosquitoes likely associate geosmin with microbes, including cyanobacteria consumed by larvae [2], who also find geosmin—as well as geosmin-producing cyanobacteria—attractive. Using *in vivo* multiphoton calcium imaging from transgenic *PUb-GCaMP6s* mosquitoes, we show that *Ae. aegypti* code geosmin in a qualitatively similar fashion to flies, i.e., through a single olfactory channel with a high degree of sensitivity for this volatile. We further demonstrate that geosmin can be used as bait under field conditions, and finally, we show that geosmin, which is both expensive and difficult to obtain, can be substituted by beetroot peel extract, providing a cheap and viable potential mean for mosquito control and surveillance in developing countries.

## RESULTS AND DISCUSSION

### Geosmin Mediates Oviposition Site Selection and Larval Attraction

**Figure 1.**
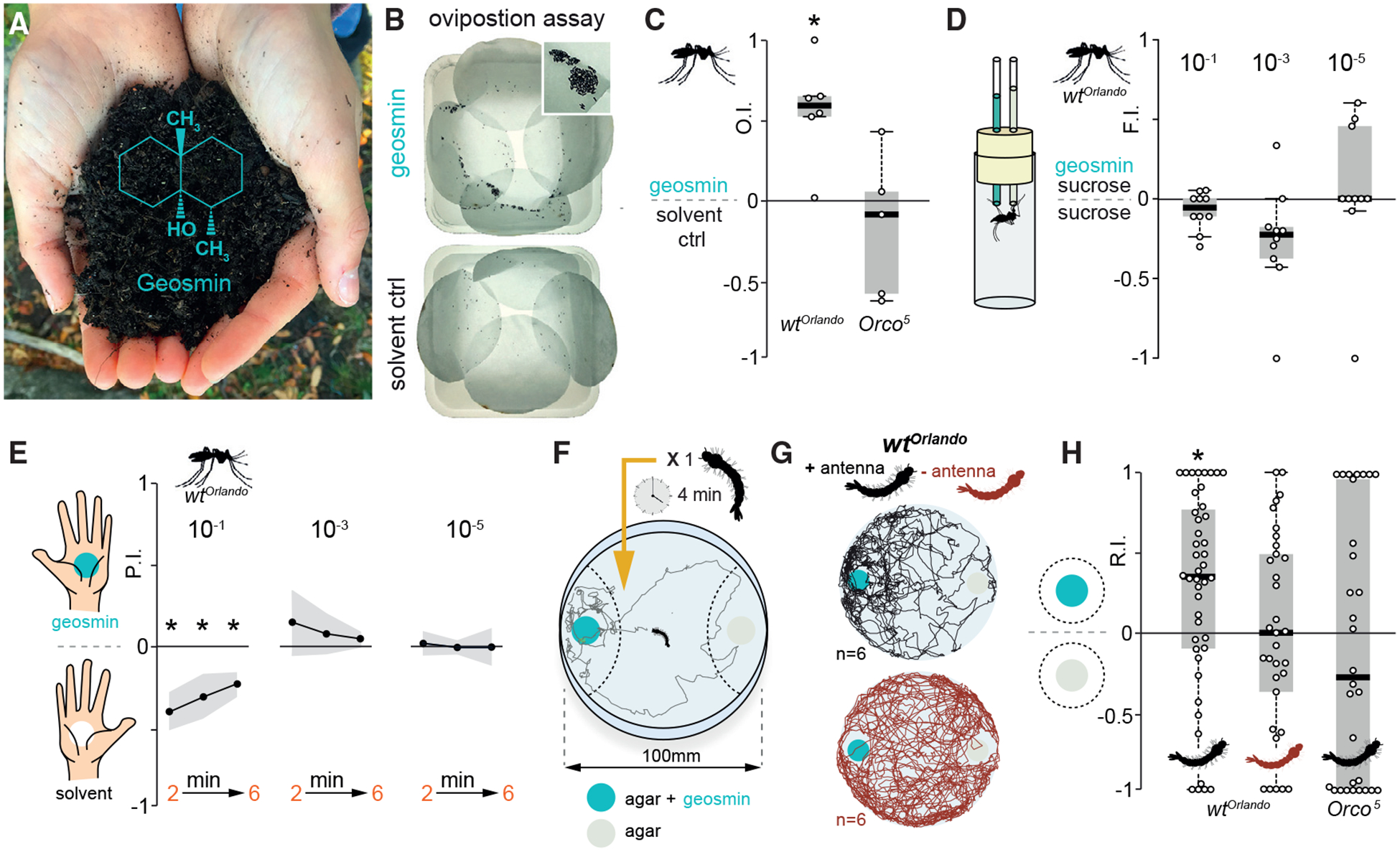
Geosmin Mediates Egg Laying Selection in *Aedes aegypti* (A) Geosmin has, to the human nose, the distinct smell of wet soil and is produced by a wide range of micro-organisms, both terrestrial and aquatic. Photo: M. Stensmyr (B) Plastic trays lined with filter paper used in oviposition experiments. On top, water with geosmin added is shown; on the bottom, water only control is shown. In the inset, close-up of a cluster of *Aedes* eggs in the geosmin-containing tray is shown. (C) Oviposition indices (OI) of wild-type (WT) (20 mosquitoes per trial; n = 6) and *Orco*^*5*^ mosquitoes (20 mosquitoes per trial; n = 6 trials) from a binary-choice test between water and water spiked with geosmin. Total number of eggs is as follows: WT 4,036, *Orco*^*5*^ 582; WT geosmin 593 ± 143 eggs, control 191 ± 58; *Orco*^*5*^ geosmin 167 ± 37, control 124 ± 23 (mean ± SEM). The edges of the boxes are the first and third quartiles, thick lines mark the medians, and whiskers represent data range. Preference was tested with one-sample Wilcoxon test, theoretical mean 0. Star denotes significantly different from 0; p < 0.05. (D) Feeding indices (FIs) from a CAFE assay of WT (n = 10) and *Orco*^*5*^ mosquitoes (n = 10) given a choice to feed from two capillaries with sucrose water (10%), one of which in addition containing geosmin (10^−1^, 10^−3^, or 10^−5^). Boxplots and statistics as per (C) are shown. (E) Probing index (PI) from WT mosquitoes (20 mosquitoes per trial; n = 5) in a constrained contact assay over 6 min, provided with a choice to approach and probe two hands (from the same individual), one of which scented with geosmin (10^−1^, 10^−3^, or 10^−5^). Shaded line indicates SEM. Statistics as per (C) are shown. (F) Schematic of the larval behavioral assay. Dashed lines denote the two zones in which time spent was measured. (G) Sample tracks of WT larvae with antennae (above) and with antennae removed (below). (H) Response indices of WT larvae with antennae (n = 44), without antennae (n = 33), and *Orco*^*5*^ mutants (n = 30) toward geosmin (10^−5^). Boxplots and statistics as per (C) are shown.

To find oviposition sites, mosquitoes rely on a combination of hygrosensation and olfaction, with the latter used to sense volatiles produced by aquatic microbes, which together with plant detritus serves as food for the larvae [3]. Microbes generate a plethora of volatile chemicals, of which several have been shown to mediate oviposition site selection in mosquitoes (e.g., [4, 5]), whereas others induce avoidance [6]. Microbial volatiles can accordingly be used to manipulate oviposition behavior in mosquitoes. Geosmin is a volatile compound produced by a wide range of micro-organisms, including taxa that inhabit typical mosquito breeding sites [2, 7]. To the human nose, this chemical has an immediately recognizable, and quite pleasant, smell of wet soil (Figure 1A). To *Drosophila melanogaster*, however, geosmin signal the presence of harmful microbes and is innately aversive [1]. Interestingly, the olfactory system of both humans and flies are extremely sensitive to geosmin [1, 8], with flies having a dedicated olfactory channel mediating information regarding this chemical [1]. How mosquitoes, including *Aedes aegypti*, perceive this important microbial smell remains, however, unknown.

In *D. melanogaster*, geosmin negatively affect egg laying preference [1]. We thus first examined whether geosmin also affects oviposition preference in the mosquito. Female mosquitoes provided with a choice to oviposit in containers with water, or water spiked with geosmin (10^−5^ dilution), preferred to lay eggs in the latter (Figure 1B). Thus, in contrast to *D. melanogaster*, *Ae. aegypti* evidently perceive geosmin as attractive. Insects detect odors via members of two large gene families: odorant receptors (ORs) [9, 10] and ionotropic receptors (IRs) [11]. The egg laying preference toward geosmin is mediated by the olfactory system, because assays with *Orco*^*5*^ mutant *Ae. aegypti* [12]– a co-receptor needed for proper OR function [13]– revealed no difference in egg numbers between water and water treated with geosmin (Figure 1C). Other behaviors, however, were barely or only moderately affected by the presence of geosmin. Mosquitoes presented with a choice of sucrose water (10%) versus sucrose water mixed with geosmin in a capillary feeder (CAFE) assay [14] (Figure 1D) showed a slight aversion (at 10^−3^ dilution) to feeding from geosmin-spiked capillaries (Figure 1D). Addition of geosmin in a constrained contact assay showed no negative effects on host attraction at moderate concentrations and modest aversion at 10^−1^ dilution (Figure 1E) (see STAR Methods).

We next examined how *Aedes* larvae react to the presence of geosmin. To address this issue, we devised a larval two-choice assay, which allowed us to monitor the position of single larvae over time (Figure 1F). Third and fourth instar *Ae. aegypti* larvae were attracted to geosmin (10^−5^), although with some individual variation (Figures 1G and 1H). As with the adults, this behavior was dependent upon olfaction, because larvae with ablated antenna showed no preference (Figures 1G and 1H), and, more-over, dependent upon the activation of *Orco*-positive neurons (Figure 1H). In summary, geosmin mediates oviposition site selection in *Ae. aegypti* and olfactory-guided positive chemokinesis [15] in larvae.

### Geosmin Producing Cyanobacteria Mediates Oviposition and Larval Attraction

**Figure 2.**
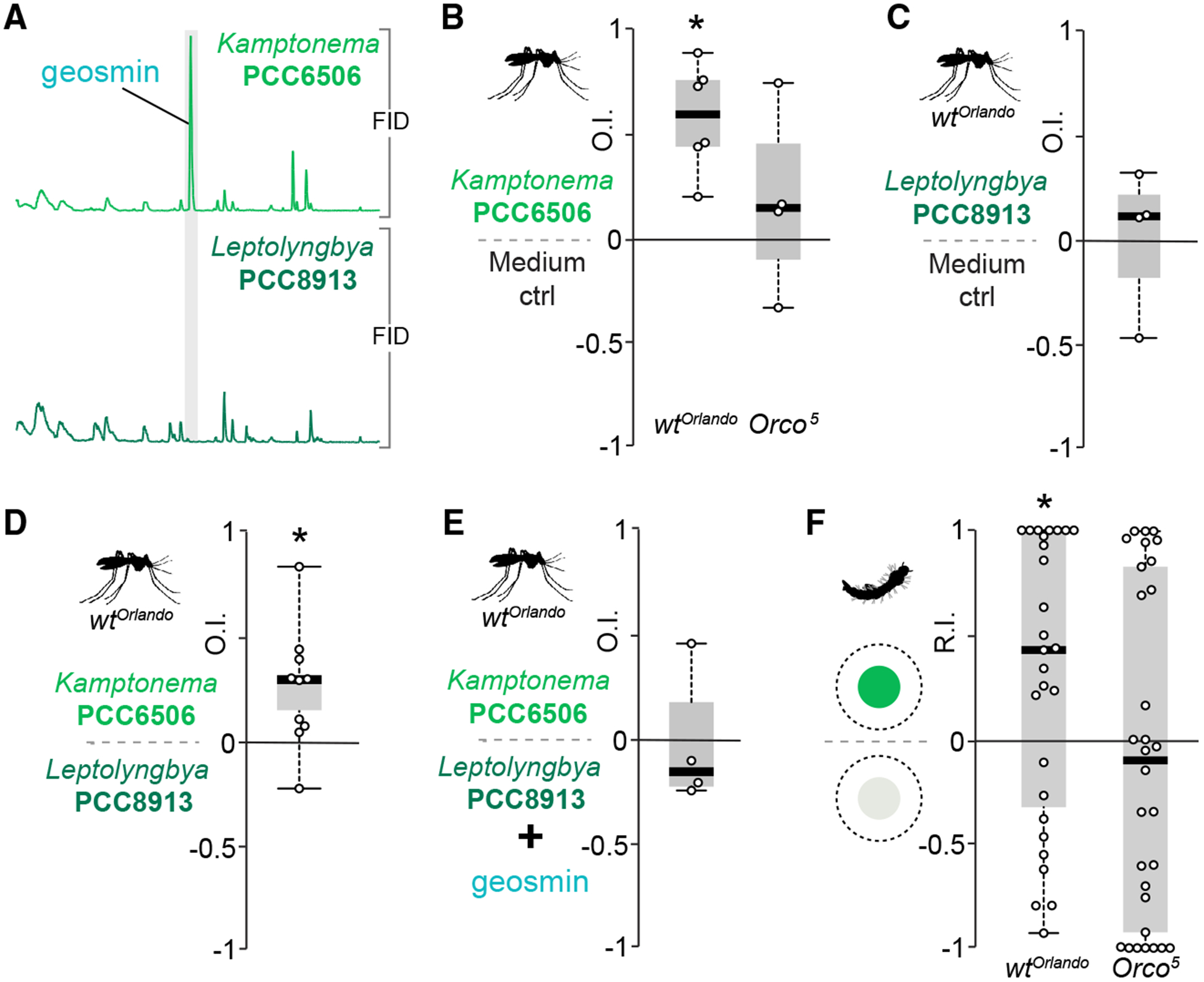
Geosmin-Producing Cyanobacteria Mediate Egg Laying and Larval Attraction (A) Flame ionization detection (FID) traces from a gas chromatography-mass spectrometry analysis of head space volatiles emitted by two strains of cyanobacteria. (B) Oviposition indices (OIs) of WT (20 mosquitoes per trial; n = 6) and *Orco*^*5*^ mosquitoes (20 mosquitoes per trial; n = 4) from a binary-choice test between growth medium and growth medium with a cyanobacteria strain (PCC6506) producing geosmin. Total number of eggs is as follows: WT 4,191, *Orco*^*5*^ 1,743; WT PCC6506 532 ± 223 eggs, control 166 ± 103; *Orco*^*5*^ PCC6506 242 ± 110, control 194 ± 92 (mean ± SEM). Boxplots and statistics as per Figure 1C are shown. (C) OI of WT mosquitoes (20 mosquitoes per trial; n = 4) from a binary-choice test between growth medium and growth medium with a cyanobacteria strain (PCC8913) not producing geosmin. Total number of eggs is as follows: 1,185; PCC8913 157 ± 40 eggs, control 140 ± 22 (mean ± SEM). Boxplots and statistics as per Figure 1C are shown. (D and E) OIs of WT mosquitoes (20 mosquitoes per trial; D, n = 4; E, n = 10) from a binary-choice test between PCC6506 and PCC8913, without (D) or with (E) geosmin (~10^−7^) added to the latter. Total number of eggs is as follows: (D) 5,112, (E) 6,445; (D) PCC6506 308 ± 80 eggs, PCC8913 203 ± 62; (E) PCC6506 822 ± 234, PCC8913+geosmin 789 ± 75 (mean ± SEM). Boxplots and statistics as per Figure 1C are shown. (F) Response indices from larvae (WT, n = 27; *Orco*^*5*^, n = 32) given a choice between agar mixed with growth medium and agar with PCC 6506. Boxplots and statistics as per Figure 1C are shown.

A plausible assumption would be that geosmin signals the presence of microbes to *Ae. aegypti*, akin to its function in flies [1], albeit with opposite valence. In the habitats of the aquatic larvae, cyanobacteria are one source of geosmin and have also been isolated from the gut of wild mosquitoes [2, 16]. We first examined how adult *Ae. aegypti* react to cyanobacteria. We selected a potentially geosmin-producing strain, *Kamptonema* sp. PCC 6506 [17], verified geosmin production via solid-phase microextraction (SPME) and gas-chromatography mass spectroscopy (GC-MS) (Figure 2A), and then performed oviposition choice experiments with wild-type *Ae. aegypti* females. Water inoculated with cyanobacteria was clearly preferred over water with only growth medium added (Figure 2B). This preference was dependent upon activation of *Orco*-expressing neurons (Figure 2B).

As evident from the GC-MS profile (Figure 2A), PCC 6506 produces, in addition to geosmin, a range of other volatile chemicals, which begs the question whether or not geosmin alone mediates the preference. To address this issue, we selected another cyanobacterial strain isolated from a mosquito breeding site (*Leptolyngbya* sp. PCC 8913) [16] not producing geosmin, as verified via SPME and GC-MS (Figure 2A). We then performed the same oviposition choice experiments as with PCC6506. The female mosquitoes now displayed no preference for the cyanobacteria-containing vessels over control (Figure 2C). Mosquitoes provided an oviposition choice between PCC6506 and PCC 8913 showed preference for the former (Figure 2D). We next provided the mosquitoes with a choice of PCC 6506 against PCC 8913, the latter spiked with geosmin, at an amount (~10^−7^) roughly equivalent to the release of geosmin from PCC 6506 as determined by SPME/GC-MS (data not shown). Mosquitoes confronted with this choice showed no preference either way (Figure 2E). We then examined how larvae react to the presence of cyanobacteria. Larvae screened in the same two-choice larval assay as before showed an overall preference to the side baited with PCC 6506 (Figure 2F). Similar to the egg laying behavior of the adults, the larval positional preference was also dependent upon *Orco*-expressing neurons (Figure 2F). We conclude that mosquitoes preferentially lay eggs in water containing cyanobacteria-producing geosmin. This preference is also observed in larvae, which presumably associate geosmin with the presence of food.

### Two-Photon Imaging Reveals Robust Neural Coding of Geosmin

**Figure 3.**
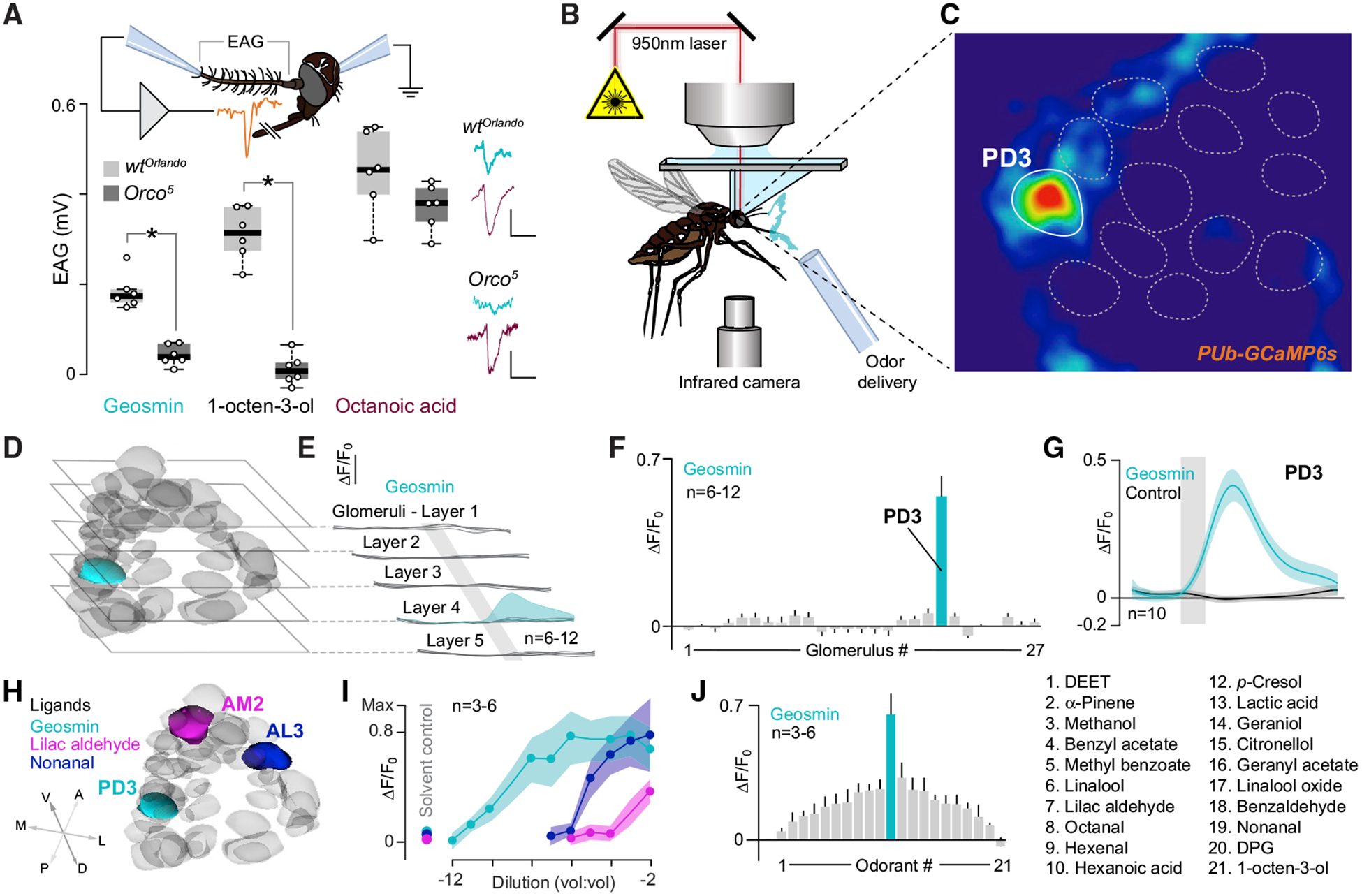
Geosmin Elicits Robust Responses in the Aedes Antennae and AL (A) Schematic of the electroantennogram (EAG) preparation (top). EAG responses from WT and *Orco* mutants to stimulation with 10^−3^ dilutions of geosmin, 1-octen-3-ol, and octanoic acid are shown. On the right, representative recordings are shown. Vertical scale bar: 0.25 mV; horizontal scale bar: 5 s. Statistical difference was measured via a Student’s t test. Star denotes significant difference (p < 0.05). (B) Schematic of the two-photon setup used to record calcium dynamics in the mosquito antennal lobe (AL). (C) Pseudocolor plot from a single preparation of ΔF/F_0_ calcium responses (0 to 1 scale) to geosmin (10^−3^ dilution), at a depth of 75 mm from the surface of the AL. Geosmin evoked a strong response in one glomerular region of interest (highlighted in white). (D) Non-responsive AL glomeruli (gray) and the geosmin-responsive glomerulus (green; the third posterodorsal glomerulus [PD3]) tentatively registered and mapped to an AL atlas and cross-referenced to a previously published atlas [19]. (E) Glomerular responses (ΔF/F_0_) to geosmin characterized at five depths (15, 30, 50, 75, and 90 μm) from the ventral surface of the AL. Each trace is the mean of one glomerulus; the PD3 response is shown in green. Vertical scale bar: 0.4%. Grey bar denotes stimulus duration (2 s). (F) Responses to geosmin across all sampled glomeruli; only the PD3 glomerulus (bar in green) showed significant calcium dynamics to geosmin compared to the solvent control (Kruskal-Wallis test: p < 0.05). Glomerular nos. 1–27 were tentatively mapped to PM1, PM2, V1–3, AM2–5, AL3, LC2, LC1, AC1, AL1, AL2, PL2, MC2, PL4, PD1, PD3, PC1, MD1–3, PD4, PD2, and AD1, respectively. Bars represent the mean ± SEM. (G) Dynamics of the calcium response to geosmin (green trace) and the solvent control (dipropylene glycol [DPG], black trace) for the putative PD3 glomerulus. Lines are the mean; shaded areas are the SEM. Grey bar denotes stimulus duration (2 s). (H) AL atlas showing the tentatively identified PD3 glomerulus (green), which is responsive to geosmin; the AL3 glomerulus (blue), responsive to nonanal; and the AM2 glomerulus (magenta), responsive to lilac aldehyde. (I) Concentration dependency of glomeruli tentatively identified as PD3, AL3, and AM2 to their cognate odorants (geosmin, nonanal, and lilac aldehyde, respectively). The glomeruli showed significantly different dose response curves (F_1,105_ = 21.5; p < 0.05), with the PD3 glomerulus having the lowest EC_50_ (10^−9^ concentration) compared to AL3 (10^−5^) or AM2 (10^−4^). Lines are the mean; shaded areas are the SEM. (J) Tuning curve for the PD3 glomerulus to a panel of 21 odorants, each tested at 10^−2^ concentration. See also Figure S1.

To examine how *Ae. aegypti* smell geosmin, we next performed electroantennography (EAG) from wild-type *Ae. aegypti* (*Orlando*). EAGs revealed distinct and dose-dependent baseline deflections in response to stimulation with geosmin (Figures 3A and S1A), suggesting that the antennae house olfactory sensory neurons (OSNs) tuned to this microbial volatile. In line with the oviposition experiments, EAGs from *Orco*^*5*^ mutants [12] showed no geosmin (or 1-octen-3-ol)-induced antennal responses, whereas octanoic acid, a compound detected by the IR pathway [18], induced responses no different from those obtained with the *Orlando* wild-type control (Figures 3A and S1B).

In *Drosophila*, geosmin selectively activates a single class of OSNs, which in turn expresses a receptor exclusively tuned to this compound [1]. Thus, we wondered whether *Ae. aegypti* detect geosmin with similar specificity. To address this issue, we next turned to functional imaging, using an *Ae. aegypti* knockin strain (*PUb-GCaMP6s*), which expresses the calcium-sensitive reporter GCaMP6s from the *ubiquitin* locus [20] (Figure S1C). *PUb-GCaMP6s* mosquitoes were glued to holders that permitted two-photon imaging of calcium responses in the antennal lobe (AL) [21] (Figures 3B and 3C). Imaging across the AL revealed no significant responses to geosmin in the vast majority of glomeruli (Figure 3D); however, one single glomerulus, located approximately 75 μm from the ventral surface of the AL, showed strong responses to geosmin (Figures 3C–3E). To tentatively identify and register this glomerulus and other glomerular regions of interest, we mapped our two-photon imaging results to an AL atlas [19]. Results suggest that the geosmin glomerulus was the third posterodorsal glomerulus (PD3) (Figures 3E and 3F) that demonstrated strong calcium-evoked responses to this compound that were time locked to the stimulus onset (Figure 3G). We acknowledge that the assigned glomerular names are tentative and possibly subject to change once an updated AL glomerular map of *Aedes* has been established.

To determine the sensitivity and tuning of this glomerulus, we next examined the putative PD3 responses under a range of geosmin concentrations (10^−2^ to 10^−12^) and compared to AL3 and AM2 glomeruli, which show the strongest responses to nonanal and lilac aldehyde, respectively [22] (Figure 3H). Compared to these other glomeruli and their cognate odorants, PD3 exhibited orders of magnitude higher sensitivity to geosmin (Figure 3I), with a median effective dose (ED_50_) of 1.75 × 10^−9^ and strong responses at picogram levels. By contrast, the ED_50_s of AL3 and AM2 to nonanal and lilac aldehyde, respectively, were 1.02 × 10^−5^ and 1.92 × 10^−4^. When factoring in the effects of vapor pressure, these differences become even greater: geosmin has a 100- to 500-fold lower vapor pressure than nonanal and lilac aldehyde (0.001 mmHg, compared to 0.1 and 0.532 mmHg, respectively), causing the airborne concentrations of geosmin to be even lower than that of nonanal or lilac aldehyde.

Given the putative PD3’s sensitivity to geosmin, we next examined how this glomerulus responded to a panel of different odorants, including compounds important for mosquito host detection, oviposition site selection, and those commonly used as repellents. From this panel, geosmin elicited the greatest response, with a 2- to 20-fold higher response compared to the other odorants (Figure 3J). Interestingly, odorants that elicited the next greatest responses were *p*-cresol and hexanoic acid, odorants suggested to be involved in oviposition site choice and blood host selection [23–25]. Although this glomerulus showed robust responses to geosmin, as measured by the kurtosis of the tuning curve (a measure of the peakedness of the distribution), with a value of 6.3, it lacked the tuning precision of the DA2 glomerulus of *D. melanogaster* to geosmin, which has a kurtosis value of 16.2 [1]. Nonetheless, this glomerulus response to geosmin indicates that this single olfactory circuit is biologically important for *Ae. aegypti* mosquitoes.

### Geosmin Attracts Mosquitoes to Oviposition Traps in the Field

**Figure 4.**
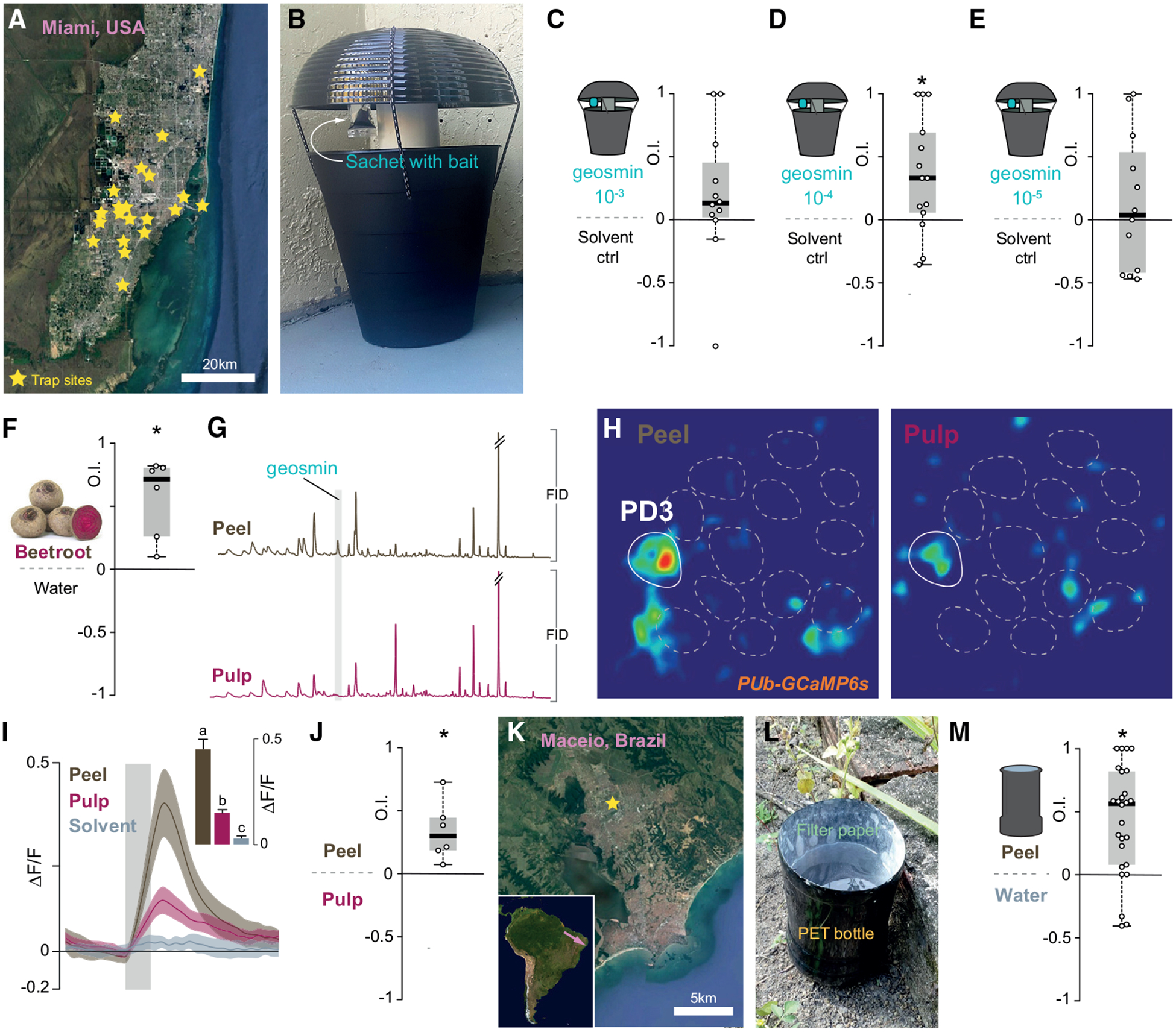
Geosmin as a Potential Mosquito Control Agent (A) Map over greater Miami area with trap sites marked. Satellite image courtesy of Google Maps. (B) Oviposition trap used for the field experiments. (C–E) OIs from Miami mosquitoes offered a choice between control traps (water only) and traps baited with geosmin. Each data point represents the average OI from a single site (n = 11–14). Total number of eggs is as follows: (C) 2,240, (D) 2,594, (E); 2,946; (C) geosmin (10^−3^) 31 ± 6 eggs per trap, control 24 ± 5; (D) geosmin (10^−4^) 35 ± 6, control 26 ± 4; (E) geosmin (10^−5^) 39 ± 8, control 31 ± 6 (mean ± SEM). Boxplots and statistics as per Figure 1C are shown. (F) OIs of WT mosquitoes (20 mosquitoes per trial; n = 6 trials) from binary-choice tests between whole beetroot extract and water. Boxplots and statistics as per Figure 1C are shown. (G) FID traces from a gas chromatography-mass spectrometry analysis of head space comparing volatiles emitted from beetroot peel and beetroot pulp. (H) Pseudocolor plot of ΔF/F_0_ calcium responses (0 to 1 scale) to beetroot peel and pulp, at a depth of 75 μm from the surface of the AL. (I) PD3 responses (ΔF/F_0_) to the extracts of the beet rind (brown), pulp (purple), and solvent (methanol) control (blue). Grey bar denotes the time course of odor stimulus. Traces are the mean; area is the SEM (n = 3 mosquitoes). Shown in the inset are mean responses to the extract. Letters denote significant differences between stimuli (Kruskal-Wallis test: χ = 63.19, p < 0.0001; multiple comparisons: p < 0.05). (J) OIs of WT mosquitoes (20 mosquitoes per trial; n = 6 trials) from binary-choice tests between whole beetroot peel and pulp. Total number of eggs is as follows: 2,878; peel 322 ± 82 eggs, pulp 158 ± 41 (mean ± SEM). Boxplots and statistics as per Figure 1C are shown. (K) Brazil field site. Satellite image courtesy of Google Maps. (L) Oviposition trap constructed from painted PET bottles lined with filter paper used for the experiments in Brazil. (M) OIs from wild Brazilian mosquitoes offered a choice between control traps (water only) and traps baited with beetroot peel extract. Each data point represents a collection event. Total number of eggs is as follows: 1,630; peel 45 ± 7 eggs, control 18 ± 3 (mean ± SEM). Boxplots and statistics as per Figure 1C are shown. See also Figure S2.

So far, we have demonstrated that geosmin mediates oviposition site preference in the laboratory. We subsequently wondered whether geosmin also works under field conditions as a potential tool for *Ae. aegypti* control. To evaluate this approach, we chose a site with high *Ae. aegypti* incidence, namely Miami (Florida, USA), where combatting mosquitoes has been a top priority since the arrival of the Zika virus in 2016 [26]. The field study was conducted across the greater Miami area at 21 sites over the course of 7 months (Figures 4A and S2), using custom-made ovitraps baited with sachets containing dilutions of synthetic geosmin (20 mL of either a 10^−3^, 10^−4^, or 10^−5^ dilution; Figure 4B). The geosmin-baited ovitraps with the 10^−4^ dilution held an increased number of eggs in comparison to control traps (Figure 4D), whereas traps baited with the higher or lower concentration did not cause an oviposition preference in comparison to water alone (Figures 4C and 4E). These results could indicate that geosmin only works within certain concentration ranges, as previously reported for other oviposition trap lures in *Aedes* [27]. Alternatively, the results might just be a consequence of the experiments being carried out at different times, under different weather conditions (Figure S2). Nevertheless, these experiments serve as proof of concept that geosmin might function in attract-and-kill mosquito control approaches. Much further work is naturally needed to establish whether geosmin-baited traps can be used to control mosquito populations.

### Geosmin Can Be Substituted by Beetroot Juice

Unfortunately, geosmin is both expensive and difficult to obtain, particularly in the developing tropical and subtropical countries where *Ae. aegypti* are causing most harm. Thus, unless a cheap source of geosmin can be identified, our findings would be of little practical consequence. Therefore, we next set out to find a more readily available source of geosmin for use in vector control. The distinct odor of geosmin is responsible for the earthy smell of beetroots (*Beta vulgaris*) [28]. Beetroots can be grown throughout much of the world and require fairly simple farming procedures. We thus wondered whether beetroot juice could be used as a substitute oviposition lure. Indeed, cups spiked with extract from beetroots contained significantly more eggs than cups with water alone (Figure 4F). We next wondered whether geosmin alone, or whether also other chemicals present in beetroot, mediate the observed preference. In this context, beetroots carry their own internal control; geosmin is reportedly produced and enriched in the peel, whereas the pulp only contains trace amounts of this compound [28, 29], which we also confirmed using GC-MS (Figure 4G).

To examine whether beetroot evoked responses in the same olfactory channel as synthetic geosmin, we again conducted calcium-imaging experiments using the *Pub-GCaMP6s* line. When stimulated with an extract of the beetroot peel, the putative PD3 glomerulus elicited strong calcium-evoked responses significantly greater than the solvent (Figures 4H and 4I). By contrast, an extract of the beetroot pulp elicited significantly lower responses compared to the peel, although still higher than the solvent control (Figures 4H and 4I). Importantly, responses to the beetroot peel were on the same order as responses to geosmin (p = 0.88) (Figure 3G). In line with the imaging results, gravid females also strongly preferred to lay eggs in cups with peel extracts over those containing pulp extract (Figure 4J). In summary, beetroot peel is a cheap and sustainable alternative to geosmin.

### Beetroot-Juice-Baited Traps Catch Mosquitoes in Brazil

Having acquired promising results with beetroots under laboratory conditions, we next conducted a small-scale field study. We performed the experiments in Northeastern Brazil (state of Alagoas; Figure 4K), which is an impoverished region with a high incidence of mosquito-transmitted infectious diseases [30]. We first devised a simple oviposition trap, constructed from used polyethylene terephthalate (PET) bottles, painted black and lined with filter paper (Figure 4L), which we placed around the campus grounds of the Federal University of Alagoas in Maceió (Figure 4K), an urban area with a high mosquito frequency. In line with the lab results, traps baited with peel extract yielded considerably more mosquito eggs than traps with water alone (Figure 4M). In short, beetroot peel works as an oviposition stimulant under field conditions and might accordingly be an inexpensive and environmentally friendly method for mosquito control in developing countries. The simple trap design can be improved, as can the beetroot formulation, to increase trap catches. Nonetheless, our findings provide an innovative and sustainable method for monitoring and potentially controlling *Ae. aegypti* in low-income areas.

### Conclusions

We show here that geosmin mediates preferential egg laying in *Ae. aegypti*, which (presumably) associates this chemical with microbes, such as cyanobacteria, present in the aquatic habitats of the larvae. *Aedes* larvae likewise find geosmin attractive, as well as geosmin-producing cyanobacteria. Using *in vivo* two-photon imaging, we find that adult *Ae. aegypti* detect geosmin with a high degree of sensitivity, with geosmin activating a single glomerulus, innervated by sensory neurons responding to geosmin already at extremely low dilutions (10^−11^). Finally, field experiments performed in Miami and Brazil with synthetic geosmin and geosmin derived from beetroot peel, respectively, demonstrate the possibility of using geosmin as bait in trap-and-kill mosquito control approaches.

The similarity by which *D. melanogaster* and *Ae. aegypti* detect and decode geosmin is striking. Both species are equipped with sensitive detection machineries for this microbial volatile. Our imaging data though suggest that the *Aedes* geosmin receptor is less selective than the *Drosophila* counterpart. The relatively broad tuning observed could, however, be a consequence of the fact that we are not only measuring calcium signals from sensory neurons but also from other cell types and/or a reflection of the fairly high stimulus concentration used. Determining the precise fashion through which *Ae. aegypti* decodes geosmin has to await identification of the receptor. A close ortholog of the *Drosophila* geosmin receptor *Or56a* is also found in the *Ae. aegypti* genome, *AeOr11* [31]. It is nevertheless intriguing that the same chemical, which appears to carry the same message, i.e., presence of microbes, induces opposing valence in these two species. How other dipterans, or other insects for that matter, react to and decode this ubiquitous compound would certainly be interesting to determine.

Many geosmin-producing microbes, including cyanobacteria, produce toxins [32]. In fact, certain strains of cyanobacteria are also acutely toxic to *Ae. aegypti* [33]. The *Kamptonema* strain used in this study produces the neurotoxin anatoxin-a (or very fast death factor) as well as the cytotoxin cylindrospermopsin [34, 35]. Possibly, mosquito larvae might have a certain degree of tolerance for cyanobacterial toxins, akin to what is found in lake flies (Chironomidae) and shore flies (Ephydridae), which habitually feed on cyanobacterial mats [36]. Not all cyanobacteria are toxic, however, and mosquitoes might be endowed with other means, olfactory and/or gustatory, to separate harmful cyanobacteria from harmless.

Apart from offering insights into how insects and mosquitoes in particular decode odors, our findings also hint at a novel and sustainable approach for mosquito control. The use of beetroot peels as bait carries the benefit that the part of the beetroot that would otherwise have gone to waste now has its distinct use. Whereas the peel can be used to trap mosquitoes, the pulp can be used to make borscht [37] or some other tasty and nourishing meal.

## STAR★METHODS

### LEAD CONTACT AND MATERIALS AVAILABILITY

Further information and requests for resources and reagents should be directed to and will be fulfilled by the Lead Contact, Marcus Stensmyr (marcus.stensmyr@biol.lu.se)

### EXPERIMENTAL MODEL AND SUBJECT DETAILS

#### Mosquito rearing

*Aedes aegypti* were reared and kept in an environmental room under LD 12:12 h cycle at 26 −28°C, 79% RH. Eggs were hatched by adding deoxygenated water with ground fish food (catalog #16152, Tetra, Melle, Germany) inside a plastic container (L: 32 × W: 17 × H: 10 cm). Post-hatching, larvae were fed daily with ground fish food. The pupae were placed in small cups with distilled water and moved to a mesh cage (L: 30 × W: 30 × H: 30 cm DP100B, Bugdorm store, Taiwan), and allowed to eclose. Adult mosquitoes were fed on 10% sucrose solution (weight: volume in distilled water) from a cotton wick inserted into a vial. Mosquitoes were blood-fed using an artificial blood feeder (CG-1836, Chemglass Life Sciences, USA) filled with defibrinated sheep blood (SB055, TCS Biosciences Ltd, Buckingham, UK) (heated to 37°C), spiked with 10 mM ATP (A1852, Sigma-Aldrich) for about 2 hours per cage. Blood-fed mosquitoes were subsequently allowed to feed on 10% sucrose solution.

### METHOD DETAILS

#### Chemical reagents

The saline solution [38], contained 150.0 mM NaCl, 25.0 mM N-2-hydroxyethyl-piperazine-N’−2-ethanesulfonic acid (HEPES), 5.0 mM sucrose, 3.4 mM KCl, 1.8 mM NaHCO_3_, 1.7 mM CaCl_2_, and 1.0 mM MgCl_2_. The pH was adjusted to 7 with 1 M NaOH. Odorants used in calcium imaging experiments were purchased from Sigma-Aldrich or Bedoukian at the highest purity (generally > 98%). Geosmin was purchased from Perfume Supply House (https://perfumersupplyhouse.com) at 10^−2^ dilution in dipropylene glycol (DPG) and from Pell Wall Perfumes (https://pellwall.com) at 10^−1^ dilution in DPG. Odorants included geosmin, terpenes: (±)linalool, lilac aldehyde (mixture of isomers), α-pinene, linalool oxide, geraniol, citronellal, geranyl acetate; aromatics: benzaldehyde, benzyl acetate, methyl benzoate, *p*-cresol, DEET; and aliphatic aldehydes, alcohols and acids: octanal, nonanal, hexenal, 1-octen-3-ol, methanol, lactic acid, and hexanoic acid. For imaging experiments, odorants were diluted 10^−2^ in mineral oil, except for geosmin and DEET, which were diluted 10^−2^ in DPG and methanol, respectively.

#### Oviposition assays

Oviposition assays were conducted to test four different stimuli and control (water): geosmin 10^−5^ (350 mL, diluted from the 10^−1^ geosmin, Pell Wall Perfumes), beetroot peel (3 g), beetroot pulp (3 g), and cyanobacteria (250 μL). 20 blood-fed mated females were used per assay. 72 h post blood-feeding, two plastic containers (L: 7 × W: 7 × H: 3 cm) filled with 80 mL distilled water were placed at opposite corners, one serving as stimulus and the other as control, inside a cage (L: 30 × W: 30 × H: 30 cm DP100B, Bugdorm store, Taiwan). Each container was lined with 5.5D Whatman filter paper (WHAT1001500, Sigma Aldrich) on the sides. Oviposition was allowed for 72 h, the number of eggs laid in each container was counted using an Olympus SZ61 stereomicroscope. Number of laid egg was compared between control and treatment, and an Oviposition Index (OI) was calculated as follows: (#_treatment_ − # _control_)/(#_treatment_ + #_control_) where #_treatment_ indicates number of eggs laid in geosmin and the #_control_ indicates number of eggs laid in control.

#### Capillary feeding assay

Capillary feeding assays were conducted to assess the effect of geosmin on nectar feeding behavior of female *Ae. aegypti*. This assay is adapted to mosquitoes based on similar assays for *Drosophila melanogaster* [14]. Starved unmated females were transferred individually to a standard polypropylene *Drosophila* rearing vial with access to two 5 mL calibrated glass capillaries embedded in cotton plugs. One of the capillaries serves as the control, containing 10% sucrose in distilled water. The stimulus capillary contained 10% sucrose spiked with geosmin (diluted in water from 10^−1^ geosmin, Pell Wall Perfumes). After two hours, the remaining liquid in all capillaries was measured, by aligning a metric ruler to the tip of the capillary and measuring the height of the liquid meniscus. A vial without mosquito was included as evaporation control. A feeding index (FI) was calculated as follows: [(stimulus − evap) − (control − evap)] / [(treatment − evap) + (control − evap)]. Vials were excluded if any of the mosquitoes died during the assay.

#### Constrained contact assay

This assay is a modification of the arm-in-cage assay, where a human hand is exposed against the mesh on the outside of the cage (L: 30 × W: 30 × H: 30 cm). 20 non-blood fed mated females were allowed to probe and “try” to feed on a human hand held at ~1.5 cm distance from the cage, enabling the mosquitoes to probe, but not to actually feed. The stimulus was a human hand with 10 μL of a geosmin dilution (in water, from 10^−1^ geosmin, Pell Wall Perfumes) applied, whereas the other hand with only solvent added served as control. Number of mosquitoes landing on the mesh and probing the hand were recorded after 2, 4, and 6 minutes. An intended biting index (BI) was calculated as follows: (#_stimulus_− # _control_) / (#_stimulus_+ #_control_) where #_stimulus_ indicates the number of mosquitoes trying to feed on the geosmin spiked hand and the #_control_ indicates number of mosquitoes trying to feed on the hand without geosmin.

#### Larval assay

*Ae. aegypti* 3^rd^ and 4^th^ instar larvae were carefully removed from rearing pans, rinsed carefully with distilled water to remove any food residues, and kept in Petri dishes with distilled water for 30 min. Odorant stock was made by dissolving a specific amount of the treatment in 2% agarose, yielding a final concentration of geosmin at 10^−5^. The assay was performed in a glass Petri dish (Ø: 10 × H: 1 cm) filled with distilled water. A test zone and control zone on opposite ends was determined and outlined. The odorant/control stock was placed into the dish 1 min beforehand to equilibrate, and an individual larva was gently introduced between the two zones. The water, odorant/control stock, and larvae, was changed after each replicate. Real time tracking was conducted throughout 4 min per replicate using Noldus Ethovision (Noldus, the Netherlands). Time spent by the larvae and the odorant/control zone was counted for each assay and a response index calculated as follows: (#_odorant_ − # _control_) / (#_odorant_ + #_control_) where #odorant indicates time larvae spent in test zone and the #control indicates time larvae spent in control zone. Respective RI values were compared with each other and analyzed for statistical significance.

#### Electrophysiology

Electroantennogram (EAG) recordings were performed using Ag-AgCl electrodes and glass capillaries filled with ringer solution (137mM NaCl; 3.6 mM CaCl_2_). Female *Ae. aegypti* were cold anesthetized for one minute before securing the body between sticky tape and dental wax. The glass capillary connected to the indifferent electrode was placed in the eye, whereas the glass capillary connected to the recording electrode was placed over the tip of the antennae. The signals were passed through a high impedance amplifier (IDAC-4, Syntech, the Netherlands) and analyzed using a customized software package (Syntech EAG-Pro 4.6, Syntech, the Netherlands). 10 μL aliquots of each dose of geosmin (diluted in water from 10^−1^ geosmin, Pell Wall Perfumes, UK) (10^−2^, 10^−3^, 10^−4^, 10^−5^, 10^−6^) was added onto a pre-cut Whatman filter paper (WHAT1001500, Sigma Aldrich) which was inserted into a sterilized Pasteur pipette. Preparation of the control stimuli 1-octen-3-ol (Sigma Aldrich) and octanoic acid (Sigma Aldrich) was done in the same manner. Stimulus pipettes were renewed for each animal tested. The stimuli were delivered via an air stream at a flow rate of 1 L min^−1^ with a puff (2 s duration) at 30 s interval. Control (water) was tested at the beginning and end of each replicate. Octanoic acid (10^−3^) and 1-octen-3-ol (10^−3^), as controls, were also tested.

#### Calcium imaging

Odor-evoked responses in the *Ae. aegypti* antennal lobe (AL) were imaged using the *PUb-GCaMPs* mosquito line. Based on immunohistochemical studies, this mosquito line shows strong GCaMP6s expression in glia, local interneurons, and projection neurons (Figure S1C, and see [22]). However, glia-like processes occurred on the exterior ‘rind’ of AL glomeruli and was restricted compared to the GCaMP labeling, thus enabling us to record from the central interior regions of the glomerular neuropil. Nonetheless, we assume the glomerular responses are a function of multiple cell types (olfactory sensory neurons, projection neurons, and local interneurons) and reflect the odor input into the system. A total of eighteen mosquitoes were used for all calcium experiments. Each mosquito was cooled on ice and transferred to a Peltier-cooled holder that allows the mosquito head to be fixed to a custom stage using ultraviolet glue. The stage permits the superfusion of saline to the head capsule and space for wing and proboscis movement [21]. Once the mosquito was fixed to the stage, a window in its head was cut to expose the brain, muscle and trachea were removed, and the brain was continuously superfused with physiological saline [38]. Calcium-evoked responses in the AL were imaged using the Prairie Ultima IV two-photon excitation microscope (Prairie Technologies, USA) and Ti-Sapphire laser (Chameleon Ultra; Coherent, USA; at 1910 mW power). Experiments were performed at different depths from the ventral surface of the AL (at 15, 30, 50, 75 and 90 μm), allowing characterization of glomerular responses to geosmin across the AL and allowing these glomeruli to be repeatedly imaged across preparations. The z-plane depths were selected to maximize the number of imaged glomeruli, while keeping repeated stimulation of the same odors to a minimum. To record odor-evoked responses, images were collected from a 110 μm × 83 μm plane at 2 Hz (line period of 1 ms), and for each odor stimulus images were acquired for 35 s, starting 10 s before the stimulus onset. Image data were imported into MATLAB (v2017; Mathworks, USA) for Gaussian filtering (2 × 2 pixel; σ = 1.5–3) and alignment using a single frame as the reference at a given imaging depth and subsequently registered to every frame to within ¼ pixel. Odor stimuli were diluted to a 1:100 concentration in mineral oil; geosmin was diluted in dipropylene glycol. During an experiment, odor stimuli were separated by intervals of 120 s to avoid receptor adaptation, and odor syringes were used once per preparation to prevent a decrease in concentration. Calcium-evoked responses are calculated as the change in fluorescence and time-stamped and synced with the stimulus pulses. After an experiment the AL was sequentially scanned at 1 μm depths from the ventral to dorsal surface to provide glomerular assignment and registration between preparations. Glomeruli (1 μm^3^ voxel) were mapped and manually registered based on the positions and odor-evoked responses of the putative AL3, MD2 and AM2 glomeruli, using an AL atlas [19] and the software Reconstruct [39].

#### Bacterial cultures

Two axenic strains *Kamptonema* sp. PCC 6506 and *Leptolyngbya* sp. PCC 8913 (obtained from the collections of the Institut Pasteur, Paris, France) were grown in BG11 media at 22°C and 5–10 μmol photon.m^−2^.s^−1^ until a concentration of around 6 × 10^7^ cells mL^−1^ had been reached, upon which the cultures were divided up in smaller aliquots and frozen (-20°C) for later use in experiments.

#### Field studies

##### Ovitraps

The custom-made ovitrap structure was mounted combining 3 pieces of white polyvinyl chloride (PVC) pipes and 2 pieces made of black plastic. The body of the trap consisted of a black bucket (Fniss trash can, black, item #602.954.38, IKEA, Sweden) where 4 holes were drilled at the top, closely to edge of the opened side. Two crossed rubber bungee cords were tied to the bucket by using the holes and were used to hold the rounded concave black lid (Camwear Round Ribbed Bowl, Item #:214RSB18CWBK, Cambro, CA, USA). A 40 cm long cylinder-shaped Ø 3 in (76.2 mm) PVC white pipe connected at the extremities to two different PVC fittings, a bottom piece (3 in × 3 in × 1–1/2 in (76.2 mm × 76.2 mm × 38.1 mm), DWV PVC Sanitary Tee Reducing, Charlotte pipe, Charlotte, NC, USA) and a top piece (Ø 3 in (76.2 mm) white slip hub #1005, Valterra, Mexico), were used to build a central pillar which was put into the center of the bucket. The bottom PVC fitting has a lateral hole that allowed the trap to be filled up with tap water from the top of the pillar. The top piece has a squared stage for supporting the lid and also holes in each corner where the scented sachet was hanged using a metal cup hook (arrow satin nickel 7/8 in (22.22 mm) cup hook, arrow utility hooks, Liberty Hardware Manufacturing Corporation, Winston-Salem, NC, USA). The half-bottom of the bucket’s inside wall was coated with a round-shaped chromatography paper (Cat n# 3030–690, GE Healthcare Life Sciences, Boston, MA, USA) as a substrate for laying. The bucket was filled up with 3 L of tap water by using the central pillar before the trap to be deployed at each site. The homemade sachets consisted of 12 cm long strips of low density polyethylene (LDPE) (2 Mil Poly Tubing Roll - 1 1⁄2’’ × 1,500’, model n. S-3521, ULINE polytubing, Pleasant Prairie, WI, USA) filled up with 20 mL of DPG (Sigma-Aldrich) for the control traps, while baited sachets were prepared with 20 mL of three different doses of Geosmin, 10^−3^, 10^−4^, and 10^−5^ (diluted from 10 ^1^ Geosmin, Pell Wall Perfumes, UK). The sachets were sealed by using a FS-300 hand sealer (FS-series, Sealer Sales, INC, CA, USA) and were kept individually inside of a Whirl-Pak® Write-On Bags - 18 oz (product number B01065WA, Nasco, Fort Atkinson, WI, USA) until placement in the traps.

##### Collections

The field experiments in Miami-Dade County (Miami, FL, USA) lasted for 30 consecutive weeks over 21 sites from August 8, 2017 to March 31, 2018 (Figure S2). For reliability purposes, we stipulated to record at least 40 positive trials (defined by the presence of at least one egg in at least one trap) for each geosmin dose tested. The 10^−4^ trials lasted for 7 weeks (from 8/11 to 10/21/2017), 10^−5^ trials for 7 weeks (from 10/28 to 12/09/2017), and 10^−3^ trials for 16 weeks (from 12/12/2017 to 03/31/2018). The custom-made ovitraps were deployed in pairs in each site. Randomly, a non-scented sachet was hung in one trap while a Geosmin-scented one was in its counterpart. The traps were setup in contact with each other (to keep similar microenvironment condition) and were exposed outside of the sites (houses, communities or apartments - no higher than the third floor) for 4 days per week. After the exposure period, the chromatography papers inside the paired traps (oviposition substrate) were collected from both control and experimental traps and were placed in respective labeled whirl pack bag until further analysis in the laboratory. The papers were qualitatively and quantitatively evaluated for mosquito egg presence under a stereo microscope (model EZ4, Leica, Germany). For species identification, the positive chromatography papers were sub-merged into deionized, deoxygenated water and larvae were fed with dissolved tablets of Tetramin tropical fish food (catalog #16152, Tetra, Melle, Germany). The emerged adults were identified by using morphological characters described in a morphological identification key [40].

##### Maceio, Brazil

The ovitraps were installed in June 2019 at the Federal University of Alagoas, Brazil (9°33’10.6’’S 35 °46’30.7’’W) over a period of 6 week. Ovitraps were made by painting 1.5 L PET bottles black and cutting the narrow opening, giving the bottles the following measurements: Ø 9 × H: 17 cm. Strips of filter paper (30 cm × 5 cm) were used to line the inside of the opening of each trap. 600 mL of water was added to each trap, with experimental traps in addition containing beet peel (10 g). Ovitrap catches were checked and collected every 3–4 days and content renewed. Collected eggs were allowed to eclose in the laboratory and adults were classified according to their morphological characteristics [40].

### QUANTIFICATION AND STATISTICAL ANALYSIS

Values are shown as boxplots; thick line shows the median, box the 25th-75th percentiles, extended by whiskers indicating 1.5x the interquartile range from the 25th-75th percentiles. All statistics were performed using R (https://cran.r-project.org/). Statistical details related to sample size and p values are reported in the figure legends, with a star denoting p < 0.05.

### DATA AND CODE AVAILABILITY

This study did not generate or analyze code. Raw data are available upon request from the corresponding author Marcus Stensmyr (marcus.stensmyr@biol.lu.se).

## Supplementary Material

Fig S1

Fig S2

## Figures and Tables

**Table:** KEY RESOURCES TABLE

REAGENT or RESOURCE	SOURCE	IDENTIFIER
Bacterial Strains		
*Kamptonema Sp*. PCC6505	Pasteur Institute, Paris, France	PCC6505
*Leptolyngpbya* Sp. PCC8913	Pasteur Institute, Paris, France	PCC8913
Biological Samples		
*Aedes aegypti Orlando*^*wt*^	DeGennaro Lab, Florida International University, Miami, USA	N/A
*Aedes aegypti Orco*^*5*^	DeGennaro Lab, Florida International University, Miami, USA	N/A
*Aedes aegypti Pub-GCaMP6s*	Riffell Lab, University of Washington, Seattle, USA	N/A
Beetroots (*Beta vulgaris*)	ICA Supermarket, Lund	N/A
Defibrinated sheep blood	Biosciences Ltd, Buckingham	SB055
Chemicals, Peptides, and Recombinant Proteins		
Geosmin 1%	Perfumery supply house	N/A
Geosmin 10%	Pell Wall Perfumes	N/A
(±) Linalool (CAS# 79-70-6)	Sigma-Aldrich	Cat#2602
Alpha-pinene (CAS# 80-56-8)	Sigma-Aldrich	Cat#147524
Linalool oxide (CAS# 60047-17-8)	Sigma-Aldrich	Cat#62141
Geraniol (CAS# 106-24-1)	Sigma-Aldrich	Cat#163333
Citronellal (CAS# 106-23-0)	Sigma-Aldrich	Cat#27470
Geranyl acetate (CAS# 105-87-3)	Sigma-Aldrich	Cat#173495
Benzaldehyde (CAS# 100-52-7)	Sigma-Aldrich	Cat#418099
Benzyl acetate (CAS# 140-11-4)	Sigma-Aldrich	Cat#50475
Methyl benzoate (CAS# 93-58-3)	Sigma-Aldrich	Cat#18344
p-cresol (CAS# 106-44-5)	Sigma-Aldrich	Cat#W233706
DEET (CAS# 134-62-3)	Sigma-Aldrich	Cat#36542
Octanal (CAS# 124-13-0)	Sigma-Aldrich	Cat#05608
Nonanal (CAS# 124-19-6)	Sigma-Aldrich	Cat#W278220
Hexenal	Sigma-Aldrich	Cat#W256102
1-octen-3-ol (CAS# 3391-86-4)	Sigma-Aldrich	Cat#W280518
Methanol (CAS# 67-56-1)	Sigma-Aldrich	Cat#34860
Lactic acid (CAS# 79-33-4)	Sigma-Aldrich	Cat#L1750
Hexanoic acid (CAS# 142-62-1)	Sigma-Aldrich	Cat#153745
Adenosine 5’-triphosphate (ATP) disodium salt hydrate	Sigma-Aldrich	Cat#A1852
D-(+)-Sucrose octaacetate	Sigma-Aldrich	Cat#252603
Potassium chloride	Sigma-Aldrich	Cat#P9333
Sodium bicarbonate	Sigma-Aldrich	Cat#S5761
Calcium chloride	Sigma-Aldrich	Cat#499609
Dipropylene glycol	Sigma-Aldrich	Cat#D215554
Methanol	Signa-Aldrich	Cat#34860
Software and Algorithms		
Syntech EAG-Pro 4.6	Syntech	https://www.dropbox.com/s/42alfiy8knzycee/SW2017.zip?dl=0
EthoVision XT	Noldus	N/A
Illustrator CC 21.02	Adobe	https://www.adobe.com/
R	R core team 2013	https://cran.r-project.org
GC/MSD ChemStation	Agilent	https://www.agilent.com/en/products/software-informatics/massspec-workstations/gc-msd-chemstation-software
MATLAB	Mathworks	https://se.mathworks.com/products/matlab.html
Reconstruct	SynapseWeb	https://synapseweb.clm.utexas.edu/software-0
NIS elements	Nikon	https://www.nikoninstruments.com/Products/Software/

## Highlights

- Geosmin mediates egg laying in the yellow fever mosquito *Aedes aegypti*
- The *Aedes* olfactory system decodes geosmin in a precise and sensitive manner
- Geosmin can be used as bait in oviposition traps
- Beetroot peel can substitute geosmin as trapping bait in low-income areas
