# Supplementary material for: Geosmin Attracts Aedes aegypti Mosquitoes to Oviposition Sites
Source: Curr Biol. Author manuscript; Available in PMC 2020 Apr 9. (PMC7144812; doi:10.1016/j.cub.2019.11.002)
Supplement: Fig S1 [file NIHMS1577436-supplement-Fig_S1.pdf]

Current Biology, Volume *30*

Supplemental Information

Geosmin Attracts *Aedes aegypti* Mosquitoes to Oviposition Sites

Nadia Melo, Gabriella H. Wolff, Andre Luis Costa-da-Silva, Robert Arribas, Merybeth Fernandez Triana, Muriel Gugger, Jeffrey A. Riffell, Matthew DeGennaro, and Marcus C. Stensmyr

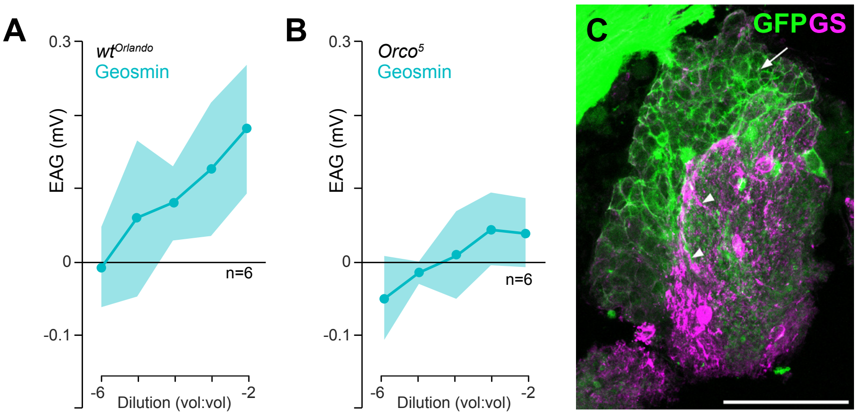

Figure S1. Neural representation of geosmin, related to Figure 3
(**A**) Electroantennogram dose response relationships from wildtype mosquitoes stimulated with increasing concentrations of geosmin. Line indicates mean respones, shaded area standard deviation.
(**B**) Same as in (A), but from *Orco*^{5} mosquitoes.
(**C**) Confocal laser scan from the antennal lobe of a *PUb-GCaMP6s Ae. aegypti* female brain labeled with antisera against glutamine synthethase (GS, magenta) and GFP (green). Arrow denote the lateral cell cluster, whereas arrowheads marks glial-like processes that form a rind around the glomeruli. Scale bar: 100 µm.

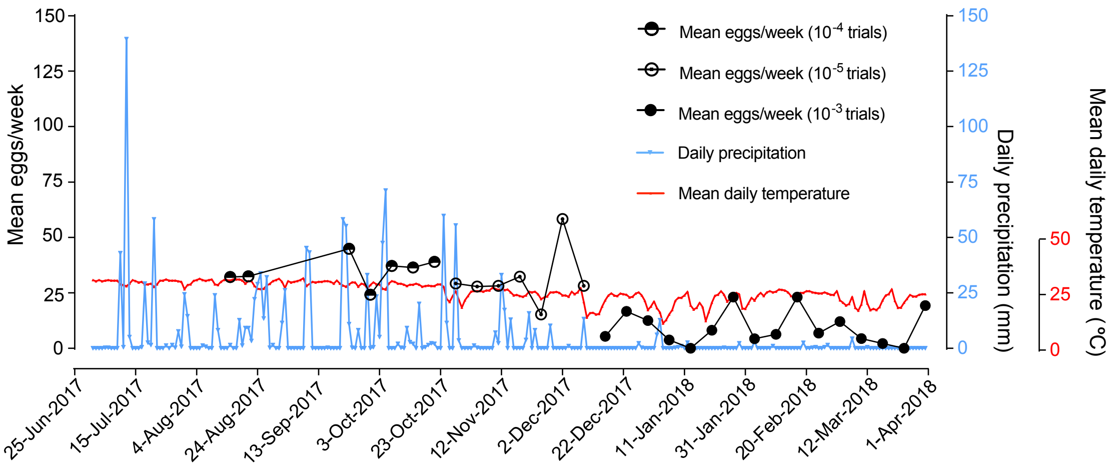

Figure S2. Trap catches and weather data for Miami, related to Figure 4
Trap catches (mean eggs/week), daily precipitation, and daily temperature during the field trapping period with geosmin in Miami, Florida, USA.
